# Brugada syndrome in a family with a high mortality rate: a case report

**DOI:** 10.1186/1752-1947-7-78

**Published:** 2013-03-18

**Authors:** Marcos Aurélio Lima Barros, Hygor Ferreira Fernandes, Cassandra Mirtes Andrade Rego Barros, Fábio José Nascimento Motta, Renata Canalle, Juan Antonio Rey, Rommel Rodríguez Burbano, France Keiko Nascimento Yoshioka, Giovanny Rebouças Pinto

**Affiliations:** 1 Genetics and Molecular Biology Laboratory, Federal University of Piauí, Av. São Sebastião 2819, Parnaíba, PI 64202-020, Brazil; 2 Marcor Clinic, Maximum Body Care and Recovery, Av. Presidente Vargas 811, Parnaíba, PI 64200-200, Brazil; 3 Research Unit, Unidad de Investigación, Hospital Universiatrio La Paz, Paseo Castellana 261, Madrid, 28046, Spain; 4 Human Cytogenetics Laboratory, Federal University of Pará, R. Augusto Corrêa 01, Belém, PA 66075-110, Brazil

**Keywords:** Brugada syndrome, *GPD1L*, High family mortality, Mutation-negative, *SCN5A*

## Abstract

**Introduction:**

Brugada syndrome is a hereditary arrhythmia characterized by a specific electrocardiographic pattern and an increased risk of sudden cardiac death, with an apparent absence of structural abnormalities or ischemic heart disease. To date, mutations in the sodium channel, voltage-gated, type V, alpha subunit gene and glycerol-3-phosphate dehydrogenase 1-like gene are estimated to account for approximately 28% of Brugada syndrome probands.

**Case presentation:**

We report the case of a 32-year-old mixed-race Brazilian man who is sodium channel, voltage-gated, type V, alpha subunit gene and glycerol-3-phosphate dehydrogenase 1-like gene mutation-negative with a type 1 Brugada electrocardiographic pattern and a history of high family mortality, including five sudden deaths among relatives of whom four were first-degree relatives.

**Conclusion:**

To the best of our knowledge, this is the first case of a patient who has Brugada syndrome and a history of sudden death in four first-degree family members. This case report reinforces the evidence that genetic studies are of limited use while determining risk but remain helpful for diagnosis, and that diagnosis via electrocardiography is of great importance in preventing adverse events and stratifying risk. Although there are several technologically advanced diagnostic tools, they might not be accessible in small towns and hospitals; however, a basic diagnostic tool like electrocardiography is easily accessible.

## Introduction

Brugada syndrome (BrS) is a hereditary arrhythmia characterized by a specific electrocardiographic (ECG) pattern and an increased risk of sudden cardiac death, with an apparent absence of structural abnormalities or ischemic heart disease. BrS has a worldwide prevalence of 1 in 2000 individuals and an autosomal dominant inheritance pattern with incomplete penetrance. The syndrome generally affects young adult men, and the first clinical symptoms normally occur at the age of approximately 40 years [1].

A definitive diagnosis of BrS is made when a type 1 Brugada ECG pattern (abbreviated as BrS-ECG+) is observed in more than one right precordial lead (V1 to V3) in the presence or absence of a sodium channel blocking agent, together with one of the following conditions: documented ventricular fibrillation, polymorphic ventricular tachycardia, family history of sudden death at an age younger than 45 years, presence of a BrS-ECG+ in family members, and inducibility of ventricular arrhythmia with programmed electrical stimulation, syncope, or nocturnal agonal respiration [2].

BrS is a disease with a strong genetic basis. Mutations in the sodium channel, voltage-gated, type V, alpha subunit (*SCN5A*) gene leading to a loss of function of the cardiac sodium channel by different mechanisms is the most common genotype found among patients with BrS. In addition to *SCN5A* alterations, mutations in the glycerol-3-phosphate dehydrogenase 1-like (*GPD1L*) gene cause abnormal trafficking of the cardiac sodium channel to the cell surface and a reduction of approximately 50% of the inward sodium current. To date, mutations in the *SCN5A* and *GPD1L* genes are estimated to account for approximately 18% to 30% and 11% to 12% of BrS probands, respectively; the prevalence of variants in other disease-related genes (such as *CACNA1C*, *CACNB2*, *SCN1B*, *KCNE3*, *SCN3B*, and *HCN4*) is yet unknown [3].

We report the case of a patient who is *SCN5A* and *GPD1L* mutation-negative BrS-ECG+ and who has a history of high family mortality, including five sudden deaths among relatives of whom four were first-degree relatives. To the best of our knowledge, this is the first case report of a patient who has BrS with a history of sudden death in four first-degree family members.

## Case presentation

**Figure 1:**
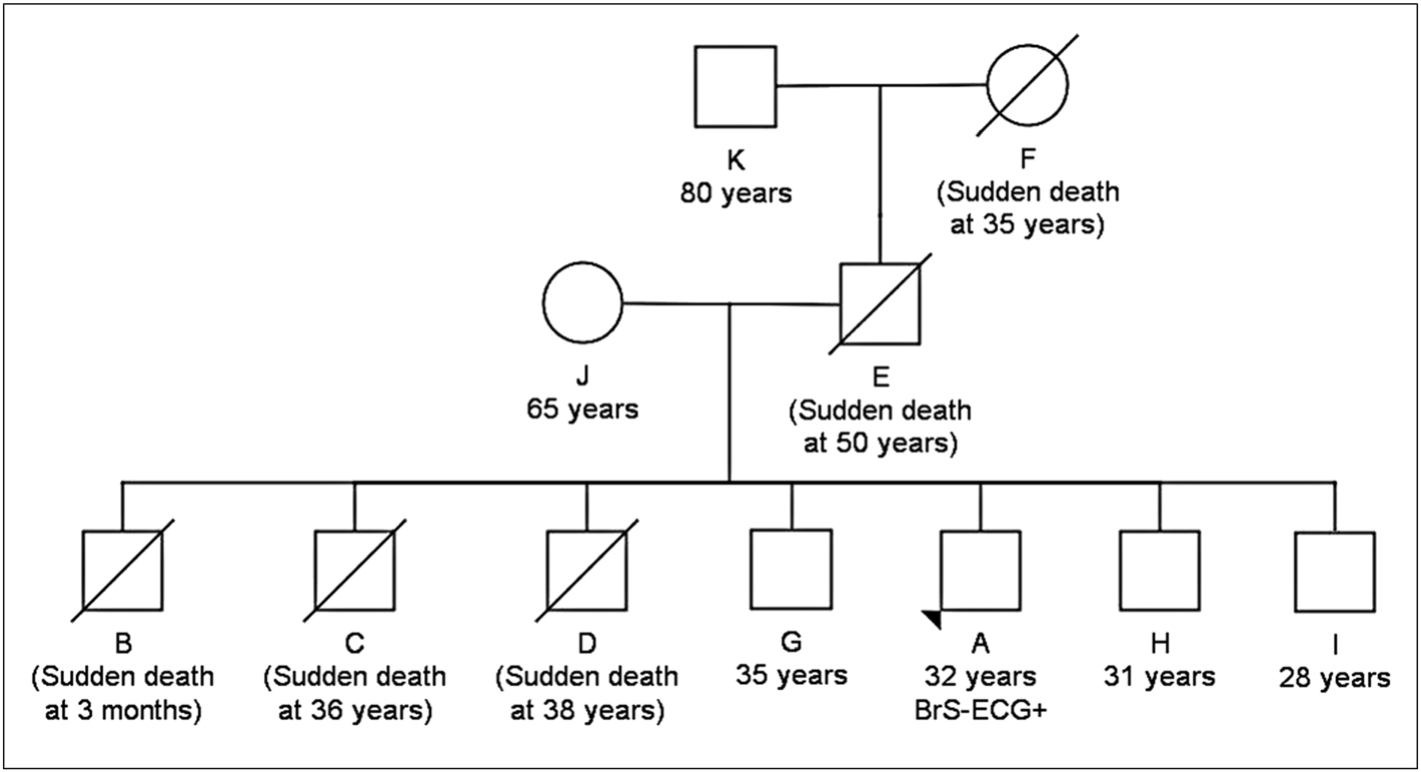
**Family pedigree.** The squares represent males and the circles represent females. Ages and age at death and/or event are in parenthesis. The proband with the Brugada syndrome phenotype (type 1 Brugada electrocardiographic pattern) is indicated by an arrowhead.

A 32-year-old mixed-race Brazilian man presented at our clinic with worry and fear of dying, given the occurrence of five sudden deaths in his family (Figure 1). The patient (A) reported that his grandmother (F), his father (E), and three of his six brothers (B, C, and D) had suddenly died in their sleep at the age of 35 years, 50 years, 3 months, 36 years, and 38 years, respectively.

**Figure 2:**
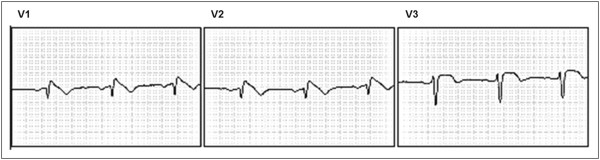
**Type 1 Brugada electrocardiographic pattern.** Electrocardiograms showing V1, V2, and V3 leads at the patient’s initial consultation.

In the evaluation of the patient and his brothers (G, H, and I), underlying structural heart disease was ruled out by results of echocardiography, chest roentgenogram, and exercise tests. In addition, acute ischemia and metabolic or electrolyte disturbances were ruled out by laboratory test results. However, the patient’s ECG result revealed spontaneous ST segment elevation in the right V1, V2, and V3 precordial leads, indicating a diagnosis of BrS (Figure 2), whereas his brothers (G, H, and I) presented normal spontaneous ECG results. The patient’s brothers refused to undergo an electrophysiological study with programmed ventricular stimulation; hence, it is possible that we have underestimated the frequency of BrS within the family.

On the basis of the patient’s ECG pattern and the knowledge of sudden death in five family members, risk stratification was performed, and according to the recommendation of the Brazilian Society of Cardiology guidelines regarding implantable electronic cardiac devices, an implantable cardioverter-defibrillator (ICD) was implanted [4]. During the 4-year follow-up period, the patient remained asymptomatic. Informed consent was obtained for mutation analysis of coding regions and splice junctions of the *SCN5A* and *GPD1L* genes.

Genomic deoxyribonucleic acid (DNA) was extracted from the patient’s peripheral blood leukocytes using standard protocols. The entire coding region and intron–exon boundaries of both genes were amplified by polymerase chain reaction, using intronic primers designed by our group from the genomic sequences of *SCN5A* (28 exons, accession number NG_008934.1) and *GPD1L* (8 exons, accession number NG_023375.1; Table 1), followed by bidirectional sequencing of amplicons using the BigDye® Terminator v.3.1 Cycle Sequencing Kit (Applied Biosystems, Forster City, CA, USA).

**Table 1:** **Intron primers used to amplify the *SCN5A *** **and *GPD1L *** **exons**

**Gene/Exon**	**Forward primer (5′ to 3′) (base pairs)**	**Reverse primer (5′ to 3′) (base pairs)**	**Product length (base pairs)**	**Annealing temperature (°C)**
***SCN5A***				
2	GAA TCA GGC CCA TTG TCT GTG (21)	GAG TTG CAC AGA AGG GTA GGC (21)	400	59
3	CAC AGT CCA AGG GCT CTG AG (20)	GAA TCA GCG CTA CTC TCA CTC C (22)	315	59
4	ATG CTG CTC AGC TTT CCT TG (20)	GGC AAA AGA GGG TAG AAG CTG (21)	282	57
5	ACG TAA GGA ACC TGG AGA ACC (21)	GCA AGG CAT AGC ACA GCA TAG (21)	236	57
6^1^ and 7^2^	GGT GGT TCT GCT TTG TAA TTC C (22)	CCC AGG CAT ATC CCT CTA GC (20)	415	57
8	CCG TGC TTG TTC TTG CCT TC (20)	GTC TGT CCT CTG TCT GGG TCT C (22)	399	59
9	CCC TCA CCA GCA TGA TGT TTC (21)	CCT GGC AGG TAA GGG AGA CAA (21)	231	59
10	GAC GGC ATG GAA CAA AGT CTC (21)	CCC ACA CTT GCT GTC CCT TG (20)	213	59
11	CCA GTG AGG GTG ACC TCT GC (20)	CAG TCA GGT GAG GGC TTA GAG G (22)	281	61
12	GCT GCA CAA AGT CTC AAT GAT G (22)	CCA CCC TGG AAA AGC TAG AAC (21)	267	57
13	GAA GAG CAC ATG TCA TGG TCG TG (23)	CAG ACC TGC TGT GGT GCC TG (20)	456	61
14	GCT GAC GCA AAT CTC CTG AT (20)	GCT TAA ATG ACC TGG GGT TG (20)	187	57
15	CTA ACT CAT TGG CTG TCC CC (20)	CTT ACC CAT GAA GGC TGT GC (20)	347	57
16	GCC AGG GAG TCT TTC CAT CC (20)	GTG ATG ACC TCA GAT TGG GTT G (22)	296	59
17	CTT CAC AAG GTC CCC TCC TC (20)	TGG GTA GAT GGA TTG ATA GAA GG (23)	488	57
18	GGA CTG GAT GGC TTG GCA TG (20)	GCT CTG GGC CCT GTA TAT GTA GG (23)	553	59
19	GCA TGG GCA GGG TCT GAA AC (20)	GCT TCA GGG ACA AAG GCA TG (20)	280	59
20	GCT ACT CAG CCC ACA CTC ACA C (22)	CTC TGG GTG GAA CTG AGG CTA G (22)	224	61
21	CAT TAG ATG TGG GCA TTC ACA G (22)	CCA GCT GGA GAC CTC CTT TC (20)	294	57
22	GCT TCA TGT CCA CCT TGT CTG (21)	CAA TGG GTT TCT CCT TCC TG (20)	252	57
23	GCC TAC TGT CTG TCC CCA AC (20)	GGC CAT AGG ACA TCA GAA GC (20)	295	59
24	GGT CTT GAA AAG GGC ATG TG (20)	CCA TTG GGA GGA AGG AAG TC (20)	386	57
25	GCT TCT GGC TTC ATC TGT CC (20)	CAG ACA CTG ATT CCC TGG TG (20)	228	57
26	CCA GCC TGT CTG ATC TCC CTG (21)	CCA ACA GGG AAG GTG AGA TGG (21)	283	61
27	CAG AAT GAG GTT GGT GCC TTC (21)	CTG GGC TGA AAG ACT GTG AAG (21)	246	57
28	CCT GCT GAG CAC TTT CCA TTT G (22)	GGT TGT ACA TGG CAT TCA GCA G (22)	396	57
29.1^3^	GCC ACC AGT AGC CAC AGT CTC (21)	GGC CTC TGG GTC AAA TTT CTC (21)	638	57
29.2	GTG CCT CTT CCA GAT CAC CAC (21)	GAT AAC CAT GGC CGA CAC CTC (21)	619	57
29.3	CCT GAG TGA GGA CGA CTT CGA (21)	GGC TGC TTT TCA GTG TGT CCT (21)	736	57
***GPD1L***				
1	ACG GTC CAG GCG GCT ACA TT (20)	GCT TCT GTG CGC TCA GTA ACG (21)	364	57
2	CTC TCC CGC CCA AGT GAG TT (20)	CCA AGT GCT GCT CAG CCA AG (20)	297	57
3	CCT TGT GGA CAG GCA AGG TT (20)	CAA TGT CTA GCC CTG GGC AG (20)	240	57
4	TCC CAT GCC TCT TTG ATT TG (20)	ACA TCA AAT GTC CAG ACT CCT GG (23)	243	57
5	GGC TGT TAT TAA TAT CCT TGT TGT C (25)	CAC AGA GCC CAT TCT TGA GGA (21)	236	57
6	GCA TCT GGG CTT TGT CAT CT (20)	CCA AGC CAG GAG TCT CAA GT (20)	322	53
7	CAG CAT AGC AAG ACC CAT TCT (21)	GGC TGG AAG TCC TGA GAA TAG (21)	299	53
8	CTG AGG TAA ATC CAG TGG CC (20)	CAT CAT GTT GCC AAG TCC TG (20)	238	53

^1^Exon 6 of *SCN5A* is only present in transcript variant 3, which is represented by the reference sequence NG_008934.1. ^2^Alternate designation 6, numbering commonly used in the literature. The same reasoning applies to the remaining exons (8 to 29). ^3^Several pairs of primers were synthesized to polymerase chain reaction-amplify exon *SCN5A* 29 owing to its large sizes.

**Figure 3:**
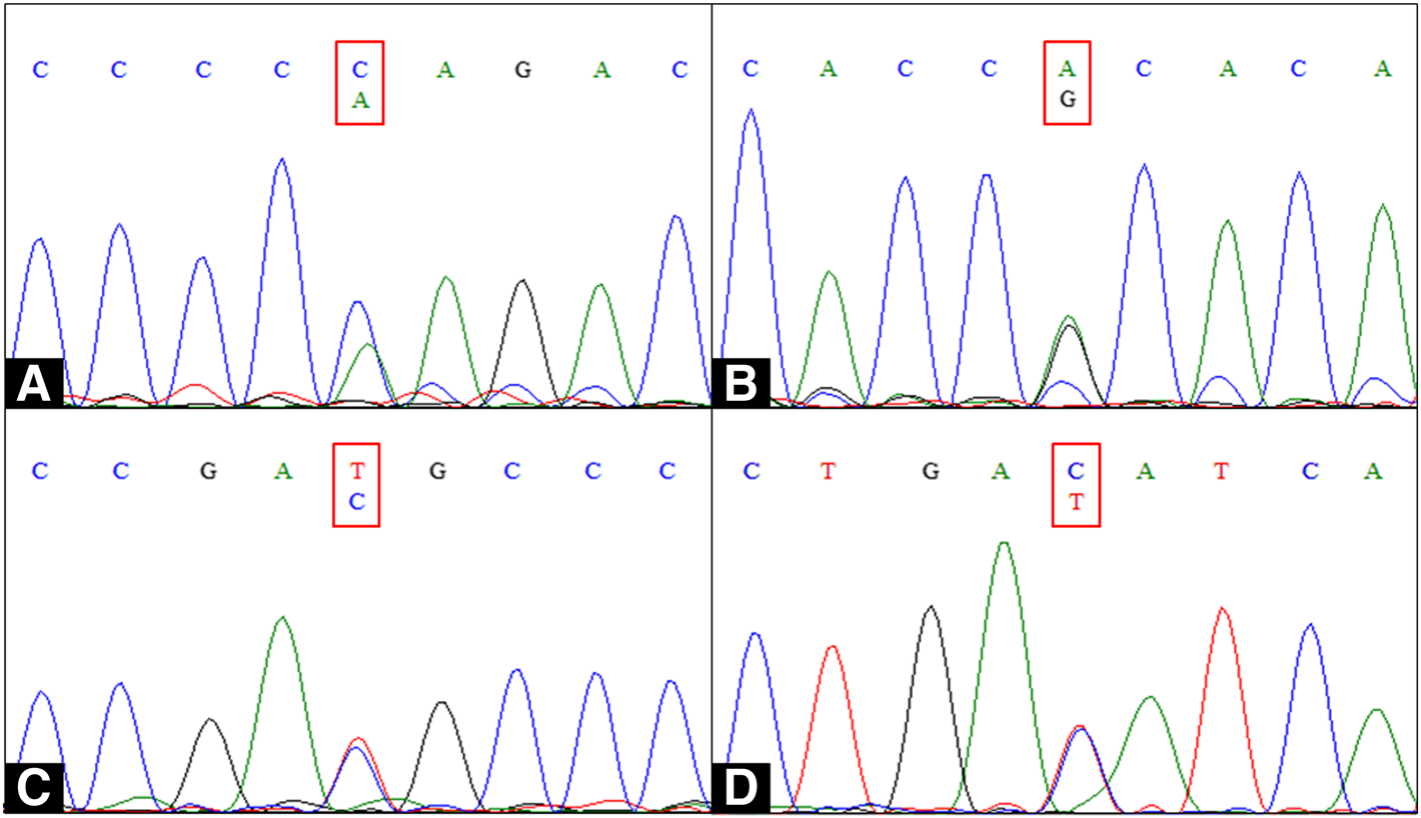
**Deoxyribonucleic acid sequencing analysis results.***SCN5A* (**A**) exon 10 IVS9-3C>A polymorphism, (**B**) exon 12 H558R, (**C**) exon 28 D1819D, and (**D**) *GPD1L* exon 4 D136D. All of the variations were heterozygous nucleotide changes in common polymorphic sites. A, adenine; C, cytosine; T, thymine; G, guanine.

We were unable to identify any causative mutation in the coding regions and intron–exon boundaries of the *SCN5A* and *GPD1L* genes. However, we identified four commonly known polymorphisms that comprised two synonymous (*SCN5A* D1819D and *GPD1L* D136D), one nonsynonymous (*SCN5A* H558R), and one splicing donor site of the intron 9–exon 10 boundary of *SCN5A* (IVS9-3C>A). All of these polymorphisms are heterozygous nucleotide changes (Figure 3).

## Discussion

BrS is a disease characterized by sudden cardiac death, typically occurring during sleep, in relatively young individuals. However, BrS has also been demonstrated in children and infants as young as 2 days and may serve as a pathogenic basis for some cases of sudden infant death syndrome. Therefore, ECG diagnosis and adequate clinical management of patients are of fundamental importance for the prevention of catastrophic events and risk stratification [5].

We present a clinical case of a patient who is mutation-negative BrS-ECG+ and who has a family history of high mortality, including five sudden deaths among relatives of whom four were first-degree relatives. The patient reported that his grandmother, father, and three of his six brothers had died suddenly in their sleep at the age of 35 years, 50 years, 3 months, 36 years, and 38 years, respectively. To the best of our knowledge, this is the first case report of a patient who has BrS with a history of sudden death in four first-degree family members. Studies reporting sudden deaths of one first-degree relative of patients with BrS are common; however, the number of reports of sudden deaths of first-degree relatives within a family decreases with the increase in number of sudden deaths within a family. García-Castro *et al*. [6], for example, reported the case of a 40-year-old man who was BrS-ECG+ whose mother and a son had died suddenly at 52 and 20 years of age, respectively. The *SCN5A* coding region and intron–exon boundaries sequencing revealed that this patient harbored an intronic mutation (IVS18-1G>A) that would affect ribonucleic acid (RNA) processing, eliminating exon 19 of the messenger RNA (mRNA) [6].

An ICD was implanted in our patient, and during the four years of follow-up, no arrhythmic event was registered. In a large meta-analysis involving 1545 BrS patients, Gehi *et al*. [7] reported that the risk of an event was increased (*p*<0.001) among patients with a history of syncope or sudden cardiac death during the follow-up period (relative risk, 3.24; 95% confidence interval [CI], 2.13 to 4.93). However, the risk of combined events during the follow-up period was higher in men who exhibited a spontaneous BrS-ECG+ than in those with a drug-induced BrS-ECG+ (4.65- and 3.47-fold, respectively). Moreover, 62% patients who recovered from an aborted sudden death event have the risk of a new arrhythmic event in the following 54 months, justifying the need for an ICD in these patients, irrespective of the presence of other risk factors [2].

Whereas the prevalence of *GPD1L* mutations is estimated to be 11% to 12%, *SCN5A* mutations are found in 18% to 30% of BrS patients, and these variations currently represent the most common BrS genotype [3]. To date, approximately 300 mutations in the *SCN5A* gene have been described in association with BrS. Six other genes (*CACNA1C*, *CACNB2*, *SCN1B*, *KCNE3*, *SCN3B*, and *HCN4*) have been associated with BrS, but the prevalence of variants in these genes is yet unknown [8].

To investigate the association between the occurrence of a BrS-ECG+ and carriership of a *SCN5A* mutation, Probst *et al*. [9] studied 13 families with a BrS-ECG+ in whom at least five family members carried the familial *SCN5A* mutation. However, a BrS-ECG+ was only found in 18% of the mutation carriers at baseline and in 61% after drug testing. Conversely, eight individuals had a BrS-ECG+ but did not carry the familial mutation. Regarding *GPD1L*, Makiyama *et al*. [10] performed an extensive screening for the prevalence of *GPD1L* mutations in 80 probands with BrS. However, only one synonymous mutation (A155A) and one intronic variant (48-30T>C), which were not present in 220 control alleles, were identified. However, the existence of family members who were carriers of the mutation but remained asymptomatic and had a normal ECG result indicates that genetic studies are of limited usefulness for determining risk but remain helpful for diagnosis [6].

As pointed out by Probst *et al*. [9], there is no reason to believe that the subjects with BrS-ECG+, who do not carry the familial gene mutation, have no true BrS, especially when these patients present spontaneous BrS-ECG+ without other clinical abnormalities to explain the ECG aspect of BrS, as observed in the patient presented in this report. This might point to the fact that loss-of-function mutations in SCN5A and GPD1L might not be sufficient to cause BrS but could act like a revelatory factor such as a sodium channel blocker challenge.

Because of its location close to the intron 9–exon 10 boundary, IVS9-3C>A could be directly functionally relevant because it could potentially induce alternative splicing. However, IVS9-3C>A and two synonymous common polymorphisms (*SCN5A* D1819D and *GPD1L* D136D) found in our patient have already been reported as polymorphisms without functional effects on the sodium channel [10].

The nonsynonymous *SCN5A* H558R polymorphism is present in 20.4% to 32% of Caucasians, 29% of people of African descent, and 10.4% of the Chinese population [11]. This polymorphism seems to be a genetic modulator of BrS among carriers of a *SCN5A* mutation, in whom the presence of the less common allele G improves the ECG characteristics and clinical phenotype [12]. In the present study, we performed genomic sequencing of the entire coding regions and intron–exon boundaries of the *SCN5A* and *GPD1L* genes; however, we are not able to exclude the possibility of a mutation in the promoter regions of these genes, which may decrease the RNA transcription rate, or a mutation in its intronic regions, which may result in an alternative splice site that competes with the normal splice sites during mRNA processing. Besides, all the genes known to be involved in the occurrence of the BrS have not been sequenced. Therefore, it is also possible that other gene mutations are responsible for the family history of high mortality observed in our case.

## Conclusion

In conclusion, we report a high mortality rate in the family of a patient who is mutation-negative BrS-ECG+; the patient’s family has a history of sudden deaths during sleep. This reinforced the hypothesis that increased vagal tone increases the incidence of ventricular fibrillation. This case report reinforces the evidence that genetic studies are of limited use while determining risk but remain helpful for diagnosis, and that diagnosis via ECG is of great importance in preventing adverse events and stratifying risk. Although there are several technologically advanced diagnostic tools, they might not be accessible in small towns and hospitals; however, a basic diagnostic tool like ECG is easily accessible. Given the possibility of sudden cardiac death from BrS, the medical community must provide information about BrS to emergency physicians, as well as to clinicians and cardiologists, indicating the need to be aware of all possible diagnoses, irrespective of how unusual they may seem.

## Consent

Written informed consent was obtained from the patient for publication of this case report and accompanying images. A copy of the written consent is available for review by the Editor-in-Chief of this journal.

## Abbreviations

BrS: Brugada syndrome; BrS-ECG+: Type 1 Brugada electrocardiographic pattern; ECG: Electrocardiographic; GPD1L: Glycerol-3-phosphate dehydrogenase 1-like gene; ICD: Implantable cardioverter-defibrillator; mRNA: Messenger RNA; RNA: Ribonucleic acid; SCN5A: Sodium channel, voltage-gated, type V, alpha subunit gene.

## Competing interests

The authors declare that they have no competing interests.

## Authors’ contributions

MALB, HFF, and CMARB examined the patient and analyzed and interpreted the patient's clinical and laboratory data. FJNM and RC performed the genome sequencing analysis. MALB, FKNY, and GRP performed the literature review and wrote the manuscript. JAR and RRB critically revised the manuscript. All authors have read and approved the final version of this manuscript.
